# Systematic review of methods for evaluating healthcare research economic impact

**DOI:** 10.1186/1478-4505-8-6

**Published:** 2010-03-02

**Authors:** Bahareh Yazdizadeh, Reza Majdzadeh, Hojat Salmasian

**Affiliations:** 1 Department of Biostatistics and Epidemiology, School of Public Health and Knowledge Utilization Research Center, Tehran University of Medical Sciences, Tehran, Iran; 2 Knowledge Utilization Research Center and Department of Biostatistics and Epidemiology, School of Public Health, Tehran University of Medical Sciences, Tehran, Iran; 3 Students' Scientific Research Center, Tehran University of Medical Sciences, Tehran, Iran

## Abstract

**Background:**

The economic benefits of healthcare research require study so that appropriate resources can be allocated to this research, particularly in developing countries. As a first step, we performed a systematic review to identify the methods used to assess the economic impact of healthcare research, and the outcomes.

**Method:**

An electronic search was conducted in relevant databases using a combination of specific keywords. In addition, 21 relevant Web sites were identified.

**Results:**

The initial search yielded 8,416 articles. After studying titles, abstracts, and full texts, 18 articles were included in the analysis. Eleven other reports were found on Web sites. We found that the outcomes assessed as healthcare research payback included direct cost-savings, cost reductions in healthcare delivery systems, benefits from commercial advancement, and outcomes associated with improved health status. Two methods were used to study healthcare research payback: macro-economic studies, which examine the relationship between research studies and economic outcome at the aggregated level, and case studies, which examine specific research projects to assess economic impact.

**Conclusions:**

Our study shows that different methods and outcomes can be used to assess the economic impacts of healthcare research. There is no unique methodological approach for the economic evaluation of such research. In our systematic search we found no research that had evaluated the economic return of research in low and middle income countries. We therefore recommend a consensus on practical guidelines at international level on the basis of more comprehensive methodologies (such as Canadian Academic of Health Science and payback frameworks) in order to build capacity, arrange for necessary informative infrastructures and promote necessary skills for economic evaluation studies.

## Background

Healthcare research can expand the frontiers of science, save human lives, and improve quality-of-life. One of the most fundamental challenges is the appropriate allocation of public and private funds to this research sector. The 2008 'Global Ministerial Forum on Research for Health' in Mali hosted ministers and representative councils from 59 countries. This forum concluded that each country should allocate 2% of Health Ministry funds to healthcare research [1]. Despite agreement that there is a need to increase funding for healthcare research, it remains difficult to convince governments and the private sector to invest in such research, especially in developing countries, which have limited financial resources. In recent years, investors and researchers have focused on the presumed benefits of healthcare research projects, so it is now necessary for health research systems to consider costs and benefits.

A reduction in healthcare research funding is likely given the current economic crisis, particularly in countries that have previously been unable to allocate sufficient funds for this purpose. In fact, before many countries can attain suitable public health standards, they will be faced with reductions in research funds, making it even more difficult to attain the required standards. The WHO 'Financial Crisis and Global Health' report emphasizes that healthcare research is not a luxury, but is rather vital for meeting the needs of the healthcare sector in times of economic crisis [2]. Therefore, we suggest that every country should evaluate the economic benefits of healthcare research to secure sufficient funds for this endeavor. The current study was designed to identify available methods of measuring the economic impact of healthcare research.

## Methods

An electronic search of English-language articles was conducted in numerous bibliographic databases in March of 2009 using a combination of keywords: health research, health system research, medical research, payback, impact, assessment, evaluation, research utilization, rate of return, internal rate of return and net present value. The databases searched were PubMed, CINAHL, Dissertation and Thesis, Urban History, Leicester, Australian Digital Thesis Program, DART-Europe E-theses Portal, and the University of Michigan's OAlster service.

An electronic search was also performed to locate 21 relevant websites (Table 1). Reports that were relevant to our study were chosen from these websites. The entire article-screening procedure was conducted independently by two individuals. Where disagreement was evident, a final decision was achieved by discussion and consultation.

**Table 1:** Sites studied

Site	Address
Canadian Institute of Health Research	http://www.cihr-irsc.gc.ca/e/193.html

Research Unit for Research Utilization	http://www.ruru.ac.uk/index.html

Organization for Economic Cooperation and Development	http://www.oecd.org/about/0,3347, en_2649_34409_1_1_1_1_1,00.html

Canadian Health Services Research Foundation	http://28784.vws.magma.ca/about/index_e.php

Industry Canada	http://www.ic.gc.ca/cgi-bin/sc_mrksv/bnkrptcy/ud/ud_srch.pl?lang=eng

ResearchResearch.com	http://www.researchresearch.com/getPage.cfm

Health Economic Research Group (HERG)	http://www.brunel.ac.uk/about/acad/herg/aboutherg

Research America	http://www.researchamerica.org/about

University of Houston System	http://www.advancement.uh.edu/impact/index.html

McCaughey Center	http://www.mccaugheycentre.unimelb.edu.au/

Academy Health	http://www.academyhealth.org/about/index.htm

English Wikipedia	http://en.wikipedia.org/wiki/Health_Impact_Assessment#Overview

Pacific Research Institute	http://liberty.pacificresearch.org/default.asp

UNC School of Public Health	http://www.sph.unc.edu/

IMS Health	http://www.imshealth.com/web/channel/0,3147,77141581_63872702_79014008,0_0.html

Yale University	http://info.med.yale.edu/womenshealth//about/index.html

University of Texas	http://www.utexas.edu/

Banner Health	http://www.shri.org/index.cfm

Health Research Council of New Zealand	http://www.hrc.govt.nz/index.html

Rural Health Research Gateway	http://www.ruralhealthresearch.org/

Primary Health Care Research and Information Service (PHC RIS)	http://www.phcris.org.au/index.php

## Results

We identified 8,416 articles in our electronic search. In primary screening that considered title and abstract (if there was an abstract), we identified 208 potentially relevant articles and subsequently requested the full texts, of which 196 were accessible. The secondary screening, which examined the full texts, yielded 18 relevant articles. We identified 11 other reports by searching Web sites (Table 1).

Some of these studies introduced theoretical definitions and frameworks, and others used practical measurements of the economic impact of healthcare research. A review of included studies indicated that evaluations of the economic impact of healthcare research were performed (a) to estimate the economic benefits of projects as a criterion for prioritizing research [3-8], and, (b) to determine the investment returns of projects (Table 2). Our study focuses on the investment returns of healthcare research projects.

**Table 2:** Selection of studies reviewed in this study

For priority setting before doing the project	Karnon J, Planning the efficient allocation of research funds: an adapted application of a non-parametric Bayesian value of information analysis,2002 [3] Townsend J, Prioritisation of health technology assessment. The PATHS model: methods and case studies,2003 [8] Coyle D, The assessment of the economic return from controlled clinical trials. A framework applied to clinical trials of colorectal cancer follow-up,2003 [5] Fleurence RL, Setting priorities for research,2004 [6] Claxton KP, Using value of information analysis to prioritise health research: some lessons from recent UK experience, 2006 [4] Fleurence RL, Setting priorities for research: a practical application of 'payback' and expected value of information,2007 [7]
**For estimation of the economic impact after doing the project**	
Macroeconomic studies	Exceptional Returns: The Economic Value of America's Investment in Medical Research.2000 [9] Exceptional Returns the Value of Investing in Health R&D in Australia,2003 [10] Exceptional Returns: The Value of Investing in Health R&D in Australia II,2005 [11] Medical Research: What's it worth? Estimating the economic benefits from medical research in the UK. Evaluation Forum,2008[12]
Case studies	Hanney S, Proposed methods for reviewing the outcomes of health research: the impact of funding by the UK's 'Arthritis Research Campaign',2004 [15] Wooding S, Payback arising from research funding: evaluation of the Arthritis Research Campaign,2005 [16] Kwan P, A systematic evaluation of payback of publicly funded health and health services research in Hong Kong,2007 [18] Wooding S, Policy and practice impacts of research funded by the Economic and Social Research Council, A case study of the Future of Workprogramme, approach and analysis,2007 [20] Nason E, Health Research- Making an Impact, The Economic and Social Benefits of HRB Funded Research,2008 [17] Kalucy L, Exploring the impact of primary health care research. Primary Health Care Research and Information Service, 2009 [19]

**Introducing the framework**	Buxton M, How can payback from health research be assessed, 1996 [13] Canadian Institutes of Health Research, Developing a CIHR Framework to Measure The Impact of Health Research,2005 [21] Kuruvilla S, Describing the impact of health research: a Research Impact Framework [23] Canadian Academy of Health Sciences, Making an Impact, A Preferred Framework and Indicators to Measure Returns on Investment in Health Research,2009 [22]
**Theoretic discussion about economic impact**	Croxson, B, Routine monitoring of performance: what makes health research and development different, 2001 [32] Peipert, J.F, The economic value of medical research: is it worth the investment, 2002 [33] Sajal K. Chattopadhyay, Economics of Prevention: The Public Health Research Agenda, 2004 [34] Michele S. Garfinkel, A Societal Outcomes Map for Health Research and Policy,2006 [35] Anthony P. Weiss, Measuring the Impact of Medical Research:Moving From Outputs to Outcomes, 2007 [36]

After reviewing the studies, we found that different methods and criteria were used to study the economic impacts of healthcare research. To measure the economic benefits of healthcare research, four questions must be answered:

1. What methods can be used to assess the economic impacts of research?

2. What economic outcomes can be attributed to research?

3. How can healthcare status be described by financial indicators?

4. When should we evaluate the economic benefits of research?

### What methods can assess the economic impacts of research?

Two methods can be used to assess the economic impacts of healthcare research; these are macroeconomics and case studies. Macroeconomic studies examine the relationship between the costs of conducting research and benefits gained at macro level and calculate the overall return but do not specify the process (in epidemiological studies; an 'ecologic study' is identical to a 'macroeconomic study'). Examples of such studies are the exceptional return reports prepared in America in 2000 [9], similar studies in Australia in 2003 and 2005 [10,11] and the study of "Medical Research: What's it worth? Estimating the economic benefits from medical research in the UK in 2008"[12].

The American report considered mortality and increased life expectancy as outcomes of research [9]. The Australian studies reviewed nationwide mortality and morbidity and the association between research investment and economic benefits. The main limitation of such studies is the presence of multiple confounding factors and the attribution problem.

In addition to showing the overall return rate, the UK study 'Medical Research - What's it worth? is an example of a study that calculates factors such as lag time, and which estimates the economic returns of individual treatments [12].

Case studies examine the impact of specific healthcare research, investigate the details of return and propose ideas for increasing it. To evaluate healthcare research in case studies, researchers have defined frameworks that classify the impact of healthcare research in various dimensions, one of which is economic impact. The frameworks identified in this study and the economic outcomes proposed are as follows:

#### The Payback framework

This framework was introduced by the "Health Economics Research Group" (HERG) of Brunel University in the UK in 1996, and was completed over several years [13-16]. One aspect of the framework was 'Broader economic benefits'. In this area, the authors defined broader economic benefits as "benefits resulting from commercial utilization of research innovations", and "benefits resulting from healthy workforce and reduction of days off work". This framework was examined in seven studies performed in the United Kingdom, Ireland, The Netherlands, and Hong Kong that were slightly modified according to local conditions and requirements [17-20]. Some studies considered 'worker stress reduction', 'public health promotion', 'mental health promotion', 'reduction of the unemployed', 'higher productions', and 'increasing equity' as economic benefits. Another study considered the following aspects as economic benefits: recruiting and keeping high-quality researchers, establishing or re-activating new companies, employing people in laboratories, increasing international funds, attracting external investment, continuing to invest in foreign companies, attracting funds for future research, introducing the country as a center-of-excellence, international recognition, facilitating access to current and available scientific recognition, identification of groups at risk for intervention, and research advancements in healthcare delivery systems and planning.

Interestingly, some of these factors carry meaningful economic benefits only at national level, and cannot be considered of significance on an international scale. All cited studies indicated the appropriateness and practicality of using the payback framework.

This approach has also been used in the design of the 'Canadian Institutes of Health Research' [21] and the 'Canadian Academy of Health Science' frameworks [22]. This illustrates that the payback framework can cover all aspects of healthcare research.

#### Canadian Institutes of Health Research framework [21]

This version of the payback framework was introduced by the 'Canadian Institutes of Health Research' in 2005. Here, the economic impacts of healthcare research are classified into four domains: commercial benefits, direct cost-savings, human capital, and the value of life and health.

The framework also describes how these domains are measured. In the commercial benefits domain, the major indicators are number and types of patents, spin-off companies and licenses for intellectual property generated, and financial returns from intellectual rights. In the direct cost-savings domain, Estimating the economic value of innovations created by health research, and (in the human capital domain), calculation of the reduction in production losses resulting from illness and/or injury, are the major indicators. In the value of life and health domain, the main indicator is the economic value of the extra years of life gained through novel treatments.

#### Research impact framework [23]

This framework was introduced in 2006. Here, also, multiple dimensions have been described for assessing the impact of research, two of which account for economic benefits. These are 'service impact' and 'societal impact'. 'Service impact' refers to cost savings in healthcare delivery systems ('limitation and effectiveness of costs'). The 'societal impact' refers to economic benefits at the macro level, such as the commercial benefits of producing and selling products, selling more effective procedures to industry, transfer of healthcare programs to the private sector, and the benefits of healthy workplaces and healthy lifestyles ('macro-economic impacts').

#### Canadian Academy of Health Sciences [22]

This framework was introduced in 2009, and has five domains that describe the impact of healthcare research. One advantage of this framework is that in addition to describing the indicators, applications are also proposed. The economic and social impacts of this framework include the following indicators:

1. Activity impact indicators:

*Labor rent or economic rent*: economic impacts (with monetary criteria) that result from employment in healthcare research rather than other sectors.

2. Commercial indicators:

◦ *Licensing returns*: money spent in obtaining licenses and/or certificates, considering the association between these materials and specific research.

◦ *Product sales revenues*: benefits of product sales, which are dependent on multiple factors.

◦ *Valuation of spin-out companies*: portfolio values of spin-out companies and sales of such companies.

◦ *Economic rent (producer rent and spillover effects)*: 'Producer rent' is the economic benefit to a company when expected revenues are exceeded. The 'spillover effect' is the impact of investment in research and development on groups that did not receive direct funding.

3. Health benefit indicators: the value of results in terms of healthcare benefits, as measured by the Quality Adjusted Life Years (QALYs) scale.

4. Well-being indicators: levels of social well-being, non-isolation of individuals, and several other indicators.

5. Social benefit indicators: extent of changes in socio-economic because of healthcare research.

Table 3 presents a summary of the economic indicators defined in the various frameworks.

**Table 3:** Indicators defined for measuring economic benefits of health research in available frameworks

Framework	Economic Benefits
Payback [13-16]	• benefits resulting from commercial utilization of research innovations
	• benefits resulting from healthy workforce and reduction of days off work

Canadian Institute of Health Research framework [21]	• commercial benefits
	• human investments
	• the value of life and health

Research impact [23]	• limitation and effectiveness of costs
	• macro-economic impacts

Canadian Academic of Health Science Framework [22]	• Research activity benefits
	• Commercial benefits
	• Health benefits
	• Well-being benefits
	• Social benefits

### What economic outcomes are attributable to research?

Healthcare research can benefit both human health and the economy, and the improvement in human health can be described by economic indicators. Multiple definitions and classifications have been proposed to explain the direct economic benefits of healthcare research, such as direct cost-savings resulting from research-driven innovation, cost-savings in service delivery systems, and benefits from commercial development of products and technologies [10,17,24]. Criteria such as death reduction, increase in lifespan, reduction of diseases, and increase in quality-of-life and life expectancy are used to assess health status. In the UK study 'Medical Research - What's it worth?' the QALYs gained and impact on the GDP of the UK (the 'spillover effect') that resulted from cardiovascular and mental health research were considered as outcomes in study of the economic impact of medical research [12].

Different methods have been proposed to identify the impact of healthcare research on human health for the purpose of differentiating it from other factors. One proposal is the study of short-term effects of specific efforts, such as cardiovascular research. Such research can include diagnostic and therapeutic studies on targeted patient populations, or preventative research on nutrition and lifestyle in apparently healthy targeted populations. The time horizon and target population should be considered in addressing the attribution problem. Thus, 'reduced mortality after myocardial infarction' may be used as an outcome of diagnostic and therapeutic research, and 'overall reduction of cardiovascular disease mortality' over an extended period of time may be used in preventative studies. The latter criterion may be influenced by behavioral changes and improved lifestyle, which lead to disease reduction, but not necessarily to reduced mortality [25].

An attribution of 50% is considered acceptable in some studies, and a sensitivity analysis of 30-70% has been used to reflect the uncertainty in the estimate [11]. However, one study showed that, with cardiovascular disease, one-third of the reduced mortality was attributable to aggressive treatments, one-third to pharmacologic developments, and one-third to behavioral changes [26]. Thus, when measuring the return of pharmacologic research on cardiovascular disease, it could be considered that only one-third of mortality and morbidity reduction may be attributed to pharmacologic developments.

### How can healthcare status be described by financial indicators?

Once the health status outcome is determined, it is necessary to describe this by use of financial indicators. Methods such as 'individual willingness-to-pay', a 'productivity approach' (e.g. additional earnings of cancer survivors [11,27], and 'maximum funds provided by the health delivery system to obtain one health unit' [17] have been used.

### When should we evaluate the economic benefits of research?

There is no general consensus on the timeframe needed to assess the economic benefits of healthcare research, but some researchers have proposed 3-5 years as appropriate [21]. After examining various studies, the 'Medical Research - What's it worth?' study in 2008 suggested 10-25 years (average: 17 years) for cardiovascular research and 9-14 years (average: 12 years) for mental health research as the average time from research to health impact [12]. However, the duration will depend on the type of study, the expected impact and the particular circumstances of each individual country.

## Discussion

Various methods and approaches are used for the economic assessment of healthcare research. There are two basic methods for studying such benefits: macroeconomic evaluation of the relationship between funds spent on research and economic benefits, and case studies which examine a single research or program.

It is often difficult to attribute an observed change in public health as causally related to funding for healthcare research, especially in macroeconomic studies. This is one of the rationales for case studies and for employing specific frameworks that facilitate the evaluation process. Among the available frameworks, the payback framework [13] has been used as a basis for all other frameworks, and has been employed more than any other model. The Canadian Academy of Health Science (CAHS) framework [22] is one novel proposed approach, the suitability and practicality of which have not yet been examined, although it does provide a more complete and comprehensive overview of the economic impact of healthcare research. It must be kept in mind that each of the available frameworks was designed for specific reasons.

A review of all relevant studies indicates that attribution of economic benefits to healthcare research requires that the impacts of interventions are measured. In fact, in such studies, research results should be attributed to a decision or change in behavior of a target group, and, next, the impact of the decision or behavioral change should be measured. Therefore, consideration of the implementation of results is very important in assessing the benefits of healthcare research [28].

Another important consideration is the cost of implementing interventions. For evaluation of the economic impact of healthcare research, two types of costs must be considered: the cost of conducting research, and the cost of implementing research results. Regarding the cost of research, combined indicators can be used to describe costs and benefit. Thus, combined indicators such as 'net present value', 'return on investment', and 'benefit-cost ratio' can be used [11]. For healthcare research to provide economic benefits, results must be implemented, but it is unclear whether the costs of implementing such results should be considered in evaluating the final economic benefits. Some researchers believe that the expense of implementing research results should be considered in evaluation of the economic benefits of such research because high expense is of little benefit for patients who are near death; research on novel care techniques has fewer benefits than research on preventative methods; and theoretical estimates have shown that, for specific gender and age groups, the benefits of prolonging life are less than curative expenditures [25]. The authors believe that it is only logical and necessary to take into account the cost of implementation of research in valuing healthcare research whose ultimate goal is to improve community's health.

In the studied reports, the sections on evaluation of the economic impacts of healthcare research are often the weakest, perhaps because of the absence of appropriate data. In fact, in most studies the researchers simply forecast economic impact, and do not actually measure outcomes. In some cases, data from other related or unrelated healthcare sectors can be used, but this information is not always available or complete. Thus, separate studies are needed to evaluate the economic benefits of healthcare research. Also, to facilitate the evaluation of the economic impacts of such research, the criteria used to define benefits and the sources of necessary information should be clearly identified from the beginning.

The indicators used to assess the economic impacts of healthcare research depend on the type of study. Thus, research projects can be classified as etiologic, interventional, policy analysis, health service, theoretical, methodological or healthcare system studies [29]. Based on the type of study, the expected economic impact, the time required to assess the impact, and comprehensive and practical indicators, should be clearly defined and specified. To facilitate economic evaluation, we suggest that researchers add sections to their reports in which the expected economic impacts are explicitly stated.

Another important issue is consideration of the benefits from domestic and foreign investment in healthcare research. Some interventions, technologies, and drugs used in a country actually result from research conducted in other countries, so not all economic benefits can be classified as domestic. The 'Medical Research - What's it worth?' study has used citations to relevant clinical guidelines to calculate the attribution of developments made in cardiovascular and mental health research in the UK to medical research worldwide [12]. The potential benefits of local investment in the research results of other countries should not be overlooked. Studies conducted to implement the research results from overseas (effectiveness studies), or investment in developing a drug that is produced in another country, can both be considered as investment in local research (the 'spillover effect').

Care must be taken in interpreting economic evaluation studies on health research. Be it positive or negative, these results may be the effect of various methodology flaws that over-represent or under-represent the true effects of research, and should be taken into account while interpreting their results. The economic benefits of healthcare research may be assessed incorrectly in the following cases: absence of valid, reliable and operational indicators for measuring economic benefits; use of incorrect and incomplete data. Even if it is proven that the investment return is low or negligible, it is still necessary to assess economic benefits, because this can indicate a waste of resources and the weaknesses should be identified using the assessment results and consequently be corrected.

## Conclusions

Healthcare research strategies have undergone significant changes over the past 20 years. Previously, 'capacity building' was a significant focus, and this led to healthcare research methodology workshops, especially in the 1980s. Next, identification of the '10/90 gap' (10% of worldwide expenditure on healthcare research is devoted to problems that primarily affect the poorest 90% of the population) led to re-prioritization of research focus[30]. Since then, the gap between research and the effects on human health have been identified, and knowledge translation was considered one of the main strategies of healthcare research in 2004, as emphasized in the Bamako Forum of 2008 [31]. Now that the world has entered an economic crisis, we suggest that it is important that the economic outcomes of research should play a pivotal role in healthcare research.

We found no reports on the research payback of low and middle income countries who have less financial resources (and allocate a smaller share of their GDP to health and its relevant researches) in our systematic review. This may partly be due to publication bias, but lack of the necessary infrastructure and skills for performing research payback studies in such countries is no doubt another reason. We suggest that international organizations, such as the World Health Organization and 'The Alliance for Health Policy and Systems Research' or other global initiatives seek to propagate, facilitate, and compare healthcare research economic evaluation throughout the world. Frameworks that have introduced more appropriate components (such as CAHS and payback frameworks) can be considered as the basis in this respect, and practical guidelines for capacity building and arranging necessary informative infrastructures should be introduced to better protect health research in these countries.

This movement should be launched with the aim of 'compiling a standard methodology on the basis of objectives, information, and available facilities in developed and developing countries', 'preparation and dissemination of tools required for measuring economic outcomes of health research', and 'specification of the expected economic outcomes'. This will help us to achieve the goals agreed upon at the Bamako Global Ministerial Forum on Research for Health in 2008.

## Competing interests

The authors declare that they have no competing interests.

## Authors' contributions

BY designed and implemented the project, and wrote the first draft of this report. RM presented the idea of the study and participated in the design and preparation of the paper. HS helped in implementing the project and conducting the systematic review. All authors read and approved the final manuscript

## Author's information

Dr Reza Majdzadeh is the Director of 'Knowledge Utilization Research Center'.
